# Robotic‐Assisted Extended Sistrunk Approach (RESA) for Salvage Surgery After Head and Neck Irradiation

**DOI:** 10.1002/hed.70343

**Published:** 2026-06-05

**Authors:** Bartosz Wojtera, Avinash Beharry, Karma Lambercy, Pascaline Dübi, Jelena Todic, Christian Simon

**Affiliations:** ^1^ Department of Otolaryngology—Head and Neck Surgery Centre Hospitalier Universitaire Vaudois (CHUV), University of Lausanne (UNIL) Lausanne Switzerland; ^2^ Department of Head and Neck Surgery, Greater Poland Cancer Centre Poznan University of Medical Sciences (PUMS) Poznan Poland

## Abstract

**Background:**

The robotic‐assisted extended “Sistrunk” approach (RESA) is a minimally invasive technique providing access to hypopharyngeal and laryngeal structures via a submental incision and vallecular pharyngotomy, bypassing the base of tongue.

**Methods:**

We report 10 RESA procedures in previously irradiated head and neck cancer patients, including six organ‐preservation surgeries and four total laryngectomies.

**Results:**

RESA enabled completion of the planned surgery, achieving R0 margins in all cases. Postoperative bleeding occurred in four patients (three Clavien–Dindo IIIb; one II). One cervical abscess was managed endoscopically. After total laryngectomy, three patients developed a subclinical pharyngeal dehiscence visualized on barium swallow exam, without evidence of a pharyngo‐cutaneous fistula. Disease‐specific survival was 100% (median 15.5 months); two patients died of unrelated causes. Swallowing improved or remained stable in seven patients at 1 year (three developed recurrence, a second primary, or died).

**Conclusion:**

RESA is a feasible salvage option with acceptable oncologic and functional outcomes in selected patients.

## 1 Introduction

Minimally invasive surgery has transformed the management of malignancies of the upper aerodigestive tract by reducing access‐related morbidity while preserving oncologic adequacy. Transoral robotic surgery (TORS) represents a major step in this evolution and has demonstrated favorable oncologic and functional outcomes, particularly in oropharyngeal cancer [1, 2, 3, 4, 5, 6, 7]. Over time, transoral robotic techniques have been applied to selected hypopharyngeal and laryngeal procedures, including partial and total laryngectomies [8, 9, 10, 11, 12].

However, the applicability of transoral approaches is fundamentally constrained by access geometry. Tumors located inferior to the tongue base and hyoid bone often remain difficult to access because of limited exposure imposed by individual anatomy, restricted mandibular excursion, tongue base bulk, or prior surgical or radiotherapeutic interventions [13, 14, 15, 16, 17, 18, 19]. As a result, a substantial proportion of patients are excluded from minimally invasive surgery despite potentially favorable oncologic characteristics, highlighting the need for alternative access strategies beyond the transoral corridor.

To address these limitations, we developed the robotic‐assisted extended “Sistrunk” approach (RESA), a remote‐access minimally invasive surgical paradigm that bypasses the transoral route entirely [18]. RESA provides direct access to hypopharyngeal and laryngeal structures through a submental incision, central hyoid resection, and vallecular pharyngotomy, enabling exposure that is independent of tongue base anatomy. This access strategy allows controlled en bloc resection with secure vascular management and has demonstrated favorable functional recovery [19]. Importantly, RESA redefines surgical exposure rather than extending transoral techniques, thereby expanding the spectrum of tumors amenable to minimally invasive resection.

Radiotherapy, with or without concomitant systemic therapy, is widely used as an organ‐preserving strategy in head and neck cancer (HNC) [5, 20, 21]. Nevertheless, locoregional recurrence and second primary tumors remain common, often necessitating salvage surgery [22, 23]. Although TORS has been explored as a minimally invasive salvage option with reduced morbidity compared with conventional open approaches [22, 24, 25], its feasibility remains limited in previously irradiated patients because of fibrosis, impaired exposure, and altered anatomy. In such settings, the lack of adequate transoral access frequently precludes minimally invasive intervention.

By providing direct, non‐transoral access to the hypopharyngeal and laryngeal framework, RESA offers a minimally invasive salvage strategy that overcomes these exposure constraints. In previously irradiated fields, RESA enables enhanced visualization and precise dissection while avoiding extensive cervical incisions and preserving the anterior cervical musculature. This approach may facilitate organ‐preserving salvage procedures or, when required, minimally invasive total laryngectomy with reduced access‐related morbidity.

Given the increasing use of organ‐preserving radiotherapy strategies, defining effective minimally invasive salvage options is of growing clinical relevance [5, 20, 21]. However, data regarding the feasibility and safety of RESA in previously irradiated patients remain limited [19]. The present study evaluates the outcomes of RESA in patients who previously underwent radiotherapy to the head and neck region. We report a case series encompassing a range of RESA procedures, including organ‐preserving resections and total laryngectomy.

## 2 Methods

### 2.1 Prospective Registry

Patients were enrolled in an institutional prospective robotic surgery registry with appropriate ethics approval (CHUV_2020_019_RM), in accordance with institutional guidelines based on Swiss legislation and national recommendations. The RESA procedure was proposed to patients who were candidates for minimally invasive robotic surgery based on multidisciplinary team assessment of patient‐ and tumor‐related characteristics, but in whom adequate exposure could not be achieved using transoral approaches. It was also offered to patients with endolaryngeal tumors requiring total laryngectomy, either as a salvage procedure (*n* = 3) or due to a non‐functional larynx (*n* = 1). All patients included in this case series had previously undergone radiotherapy to the head and neck region.

### 2.2 Surgical Technique

The RESA operative technique has been previously described [18, 19]. All patients underwent tracheostomy. Briefly, a submental incision is made, and a subplatysmal working space is created. The central portion of the hyoid bone is resected, followed by a pharyngotomy guided by a Langenbeck retractor placed transorally in the vallecula. This provides access to the hypopharynx and larynx while bypassing the oropharyngeal anatomy.

The principal part of the procedure is then performed using the da Vinci Xi robotic system. The robotic system is docked on the right side of the patient. Exposure is achieved using a Feyh‐Kastenbauer retractor (Olympus Gyrus Medical Inc., Tuttlingen, Germany) fixed on the mandible for vertical retraction, combined with a TrimLine cervical retractor (Medtronic Sofamor Danek Inc., Memphis, TN, USA) for lateral exposure. Dissection is performed using a 30‐degree endoscope and three instruments: monopolar curved scissors and two Maryland bipolar forceps. If instruments are oriented in a cephalic position, a robot boom rotation is done to avoid any collision with the mental prominence.

#### 2.2.1 Organ‐Preservation Surgery

Robotic organ‐preservation surgery is performed according to tumor location and includes a supraglottic partial laryngectomy and/or partial hypopharyngectomy.

Supraglottic laryngectomy begins with an incision in the vallecula, followed by resection of the pre‐epiglottic space and the entire epiglottis down to its root toward the anterior commissure. Posteriorly, resection proceeds through the aryepiglottic and ventricular folds and follows the ventricle on both sides. The upper half of the thyroid cartilage is exposed. Control of the superior laryngeal vessels is achieved by dissecting laterally to the superior cornu of the thyroid cartilage and laterally to the thyrohyoid muscle into the surrounding soft tissues.

Subsequently, a thyroid cartilage–base of tongue pexy is performed in a conventional manner. The upper half of the thyroid cartilage is resected to reduce its vertical height, and the remaining cartilage is mobilized. Three to five 0–0 or 1–0 Ethibond sutures are then placed, anchored inferiorly around the thyroid cartilage and superiorly through the base of the tongue, and tied simultaneously with the larynx in an anteriorly flexed position.

Then the infrahyoid musculature is sutured to the suprahyoid musculature. Finally, the submental incision is closed in two layers, and suction drains are placed as required.

#### 2.2.2 RESA Total Laryngectomy

Through the tracheostomy access, the trachea is partially mobilized, and the cricoid region and thyroid alae are released laterally. The prelaryngeal musculature is identified, partially divided to expose the inferior laryngeal framework, and left attached to the platysma, allowing circumferential dissection of the larynx from inferiorly up to the level of the cricoid and thyroid notch. Care is taken to secure branches of the superior thyroid artery bilaterally.

The robotic system is then docked through the RESA corridor. The piriform sinuses are released bilaterally, the thyroid cartilage, including the superior cornua, is dissected, and the larynx is circumferentially released. The pre‐epiglottic and post‐cricoid incisions are extended until they meet the tracheal dissection plane. A representative video illustrating the robotic phase of the total laryngectomy is provided as Video S1.

Neopharyngeal suturing is performed conventionally through the submental access and reinforced by layered closure of the preserved prelaryngeal musculature. The submental incision is closed in two layers, and suction drains are placed as required.

### 2.3 Rehabilitation

The swallowing and voice rehabilitation protocol has been described in detail in a previous article [19]. Briefly, postoperative rehabilitation is provided by a speech and language therapist using analytic and functional techniques, with initial goals of airway protection and tracheostomy closure. Therapy includes tongue and pharyngeal strengthening, improvement of laryngeal elevation, voice exercises to enhance glottic closure, and individualized compensatory swallowing strategies.

### 2.4 Functional Outcomes Assessment

Swallowing function was evaluated using the Functional Oral Intake Scale (FOIS) [26, 27] at predefined time points: preoperatively and postoperatively at 1 week, 1 and 3 months, and 1 year. Diet textures were progressively adapted according to functional findings. Rehabilitation continued on an outpatient basis with regular phoniatric follow‐up.

## 3 Results

### 3.1 Patients and Interventions

A total of 10 patients previously treated with RT within the head and neck were operated using the RESA technique, including six organ‐preservation surgeries and four total laryngectomies. Of these, nine procedures were performed for oncological indications and one for a non‐functional larynx. In all cases, RESA provided adequate access to conduct the planned surgery, resulting in R0 margins in all nine oncologic resections. Representative intraoperative footage of the robotic phase of RESA total laryngectomy is provided (Video S1). Overall patient characteristics are summarized in Table 1, with detailed clinical data including follow‐up and prior oncological history presented in Table 2. The median follow‐up was 15.5 months (range, 5–53 months).

**TABLE 1:** Overall patient characteristics.

Characteristic	Number
**Patients**	10
**Age**	
Median (range)	69.5 (55–90)
**Gender**	
Male	10
**Time from last irradiation to RESA**	
Median (range) (months)	10.8 (5.6–155.4)
< 2 years	9
> 2 years	1
**Indication for surgery**	
Non‐functional larynx	1
Second primary or recurrent tumor	9
**Type of RESA surgery**	
Organ‐preservation surgery	6
Supraglottic partial laryngectomy	3
Partial hypopharyngectomy	2
Partial base of tongue resection	1
Total laryngectomy	4
**Margin status**	
R0	9
**Clavien‐Dindo perioperative complications** [28]	
II	5
IIIa	1
IIIb	4
**Mayo Clinic classification hemorrage** [29]	
0	6
Minor | Intermediate	1 / 3
Hospital stay days median (range)	29.5 (22–77)
**Gastrostomy dependance**	4
Short term (< 6 months)	3
Long term (> 6 months)	1

**TABLE 2:** Detailed patient characteristics with follow‐up and previous oncological history.

Patient	Surgery information	Pexy	Hospital stay (days)	Complications	Functional outcomes	Follow‐up	Past treatment information
Pt 1, male, 63 years^a^	Second primary epiglottic SCC, cT1N0M0, pT2, Supraglottic partial laryngectomy	No pexy	29	D18: bleeding (Mayo Clinic classification: intermediate) D20: pneumonia Clavien‐Dindo IIIb	2 years following RESA: FOIS 6 PAS 2	44 months: no evidence of recurrence 42 months post‐RESA diagnosed with esophageal cancer with distant metastases	Left oropharyngeal SCC, pT2N3b, p16‐negative: left lateral pharyngectomy with LND (ENE+), followed by adjuvant RT (66 Gy for the tumor site, 59.4 Gy for the intermediate area, and 52.8 Gy for the ND low risk area) Right‐sided regional recurrence: RND followed by adjuvant CRT with Cisplatin, 66 Gy by tomotherapy and 52.8 Gy for the ND area
Pt 2,male, 90 years^a^	Second primary hypopharyngeal SCC (retro‐cricoid), cT1N1M0 pT1N1, Partial hypopharyngectomy	No pexy	42	D12: bleeding (Mayo Clinic classification: intermediate) Clavien‐Dindo IIIb	2 years following RESA: FOIS 6 PAS 1	38 months: no evidence of recurrence, died of other cancer 36 months post‐RESA patient diagnosed with the tumor of floor of the mouth, cT4N1, died 38 months post‐RESA	Right periauricular cutaneous SCC, pT2N3b (ENE+): Tumor resection with superficial parotidectomy and RND followed by adjuvant RT (51.2 Gy for the tumor site, 51.2 Gy for the metastatic lymph nodes, and 44.8 Gy for the ND low risk area) 2 Multiple previous cutaneous SCC and BCC tumors of head and neck region treated with surgical resections
Pt 3, male,68 years^a^	Recurrent base of tongue SCC, cT3N0M0 pT3, Partial base of tongue resection	No pexy	30	D12: bleeding (Mayo Clinic classification: minor) Clavien‐Dindo II	2 years following RESA: FOIS 6 PAS 1 PEG insertion 1‐month post‐RESA, PEG dependence for 4 months, then PEG removal	53 months: no evidence of recurrence 14 months post‐RESA patient diagnosed with the recurrence epiglottic SCC treated with laser CO_2_ resection	Multiple synchronous SCCs: oral cavity cT2 (soft palate) and pTis (floor of the mouth), oropharynx cT4a, and pulmonary cT1N2cM0: Tumor resection of oral cavity cancers, CRT with Cetuximab and RT with 69.96 Gy for the oropharynx primary and 66 Gy for the pulmonary lesion
Pt 4, male, 84 years	Recurrent epiglottic SCC, cT2N0M0 pT1, Supraglottic partial laryngectomy	Pexy	77	Urinary infection with sepsis Clavien‐Dindo II	3 months following RESA: FOIS 1 PAS N/A PEG insertion 2 months after RESA, PEG‐dependance until last follow‐up	7 months: no evidence of recurrence, died of COVID‐19 infection followed by sepsis	Epiglottic SCC, cT2N0M0: single‐modality RT (69.96 Gy for the high‐risk area and 52.8 Gy for the low‐risk area)
Pt 5, male, 74 years	Non‐functional larynx, severe dysphagia, Total laryngectomy	NA	23	Pharyngeal dehiscence with no external fistula, cervical abscess, drained endoscopically Clavien‐Dindo IIIb	2 years following RESA: FOIS 7 PEG insertion 3 months prior to RESA, PEG removal 9 months after RESA	29 months: no evidence of recurrence	Right false vocal cord SCC, cT2N2cM0: CRT with Cisplatin, 69.96 Gy for tumor and positive lymph nodes, 54 Gy for elective lymph node levels
Pt 6, male, 67 years	Recurrent base of tongue SCC, cT3N0M0, pT4a, Supraglottic partial laryngectomy	Pexy	22	Pneumonia Clavien‐Dindo II	1 year following RESA: FOIS 2 PAS 6	11 months: alive with local recurrence Palliative treatment in other center, no information available	Base of tongue SCC, cT4aN2cM0, p‐16 negative: CRT with Cisplatin, 69.96 Gy for the tumor, 56.1 Gy for the lymph nodes low risk area
Pt 7, male, 59 years	Recurrent right piriform sinus SCC, cT1N0M0, pT1, Partial hypopharyngectomy	No pexy	41	Cervical subcutaneous emphysema after surgical closure of tracheostomy Clavien‐Dindo IIIa	6 months following 1st RESA: FOIS 6 PAS 2 PEG insertion 1 month after RESA, PEG dependence for 4 months, then PEG removal	16 months: no evidence of recurrence 6 months post‐RESA a second primary SCC	Right piriform sinus SCC, cT4aN2bM0: 3 cycles of neoadjuvant TCF chemotherapy (docetaxel, cisplatin, 5‐fluorouracyl), followed by CRT with Cisplatin, 69.96 Gy for the tumor, 52.8 Gy for the lymph nodes
Pt 8, male, 71 years	Recurrent right supraglottic SCC, cT2N0M0, pT2, Total laryngectomy	NA	26	D18: bleeding (Mayo Clinic classification: intermediate) Clavien‐Dindo IIIb	1 year following RESA: FOIS 7 PAS N/A	15 months: no evidence of recurrence	Right supraglottic SCC, cT4bN2bM0: CRT with Cisplatin, 69.96 Gy for the tumor and metastatic lymph nodes, 52.8 Gy for the larynx and other lymph nodes
Pt 9, male, 73 years	Recurrent glottic SCC, cT4aN0M0, pT4a, Total laryngectomy	NA	34	Subclinical pharyngeal dehiscence Clavien‐Dindo II	1 year following RESA: FOIS 7 PAS N/A	13 months: no evidence of recurrence	Left vocal cord SCC, pTisN0M0: laser CO_2_ resection Left anterior vocal cord SCC, cT1aM0 and posterior glottic SCC cT1N0M0: single modality RT (54 Gy for high‐risk area and 45 Gy for low‐risk area)
Pt 10, male, 55 years	Recurrent glottic SCC, cT2N0M0 pT2, Total laryngectomy	NA	29	Subclinical pharyngeal dehiscence Superficial venous thrombosis of the upper limb Clavien‐Dindo II	3 months following RESA: FOIS 5 PAS N/A	5 months: no evidence of recurrence	Glottic verrucous SCC cT1bN0M0: single modality RT 57 Gy for high‐risk area and 52.25 Gy for low‐risk area

Abbreviations: BCC, basal cell carcinoma; CRT, chemo‐radiotherapy; ENE+, extranodal extension of lymph node metastasis; FOIS, functional oral intake scale; LND, left neck dissection; NA, not applicable; ND, neck dissection; NPWT, negative pressure wound therapy; PAS, penetration‐aspiration scale; PEG, percutaneous endoscopic gastrostomy; RIG, radiologically inserted gastrostomy; RND, right neck dissection; RT, radiotherapy; SCC, squamous cell carcinoma; VAC, vacuum assisted closure.

^a^ Patients reported also in a previous study: [19] Pt 1 = Pt 1, Pt 2 = Pt 3, Pt 3 = Pt 5. In the current study we present these cases in the context of previously irradiated head and neck and the extended follow‐up.

### 3.2 Complications

The most frequent complication was postoperative bleeding from the resection bed during the granulation phase (40%). Three of these bleeding events were classified as intermediate according to the Mayo Classification and as Clavien–Dindo grade IIIb, while one event was considered minor (Clavien–Dindo grade II) [28, 29].

Three of the four patients who underwent RESA‐assisted total laryngectomy developed a pharyngeal dehiscence. In the first case (Patient 5), a barium swallow exam was performed 2 weeks postoperatively, finding no signs of dehiscence, and oral feeding was started. However, this patient came back with a cervical abscess and a pharyngeal dehiscence confirmed radiologically 6 weeks postoperatively. The abscess was successfully managed with endoscopic drainage (Clavien‐Dindo IIIb), and the defect subsequently healed with conservative measures. Oral feeding was resumed at postoperative week 18 (Figure 1).

**FIGURE 1:**
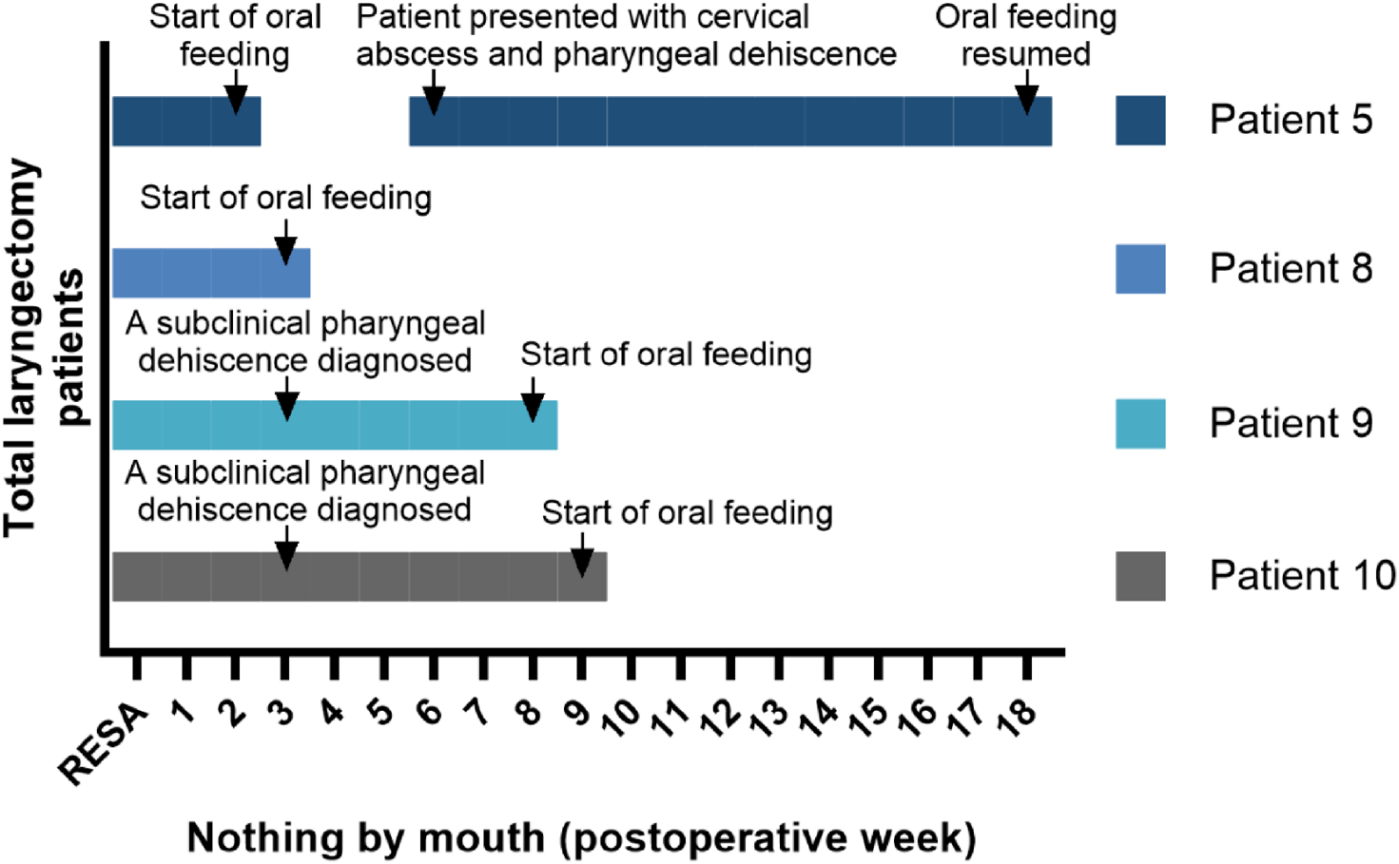
Duration of postoperative nothing by mouth (NPO) status in patients undergoing RESA total laryngectomy, illustrating time to restoration of oral feeding. [Color figure can be viewed at wileyonlinelibrary.com]

Based on this experience, the barium swallow exam was postponed to the third postoperative week in the following patients. Patient 8 started oral feeding at the third postoperative week without any complications. In both Patient 9 and Patient 10, a subclinical dehiscence was detected on barium swallow examination, and they remained on non‐oral feeding until closure with conservative management (Clavien‐Dindo II) on follow‐up imaging (Figure 1).

Other complications included pneumonia (Clavien‐Dindo II), superficial venous thrombosis of the upper limb (Clavien‐Dindo II), a urinary infection progressing to sepsis (Clavien‐Dindo II), and a wound infection after tracheotomy closure (Clavien‐Dindo IIIa)—all of which resolved with conservative treatment (Tables 2 and 3).

**TABLE 3:** Complications.

Procedure‐related complications	Clavien‐Dindo classification	*n*	%
Postoperative bleeding (intermediate [29])	IIIb	3	30
Subclinical pharyngeal dehiscence	II	2	20
Pneumonia	II	2	20
Postoperative bleeding (minor [29])	II	1	10
Pharyngeal dehiscence with cervical abscess	IIIb	1	10
Wound infection after tracheotomy closure	IIIa	1	10

Non‐procedure‐related complications		*n*	%
Superficial venous thrombosis of the upper limb	II	1	10
Urinary infection with sepsis	II	1	10

Although a pharyngeal dehiscence occurred in some patients identified via barium swallow exam, no pharyngo‐cutaneous fistula developed after RESA total laryngectomy, and no patient required an open surgical revision. Figure 2A,B presents representative postoperative neck photographs, illustrating the favorable esthetic outcomes of RESA total laryngectomy.

**FIGURE 2:**
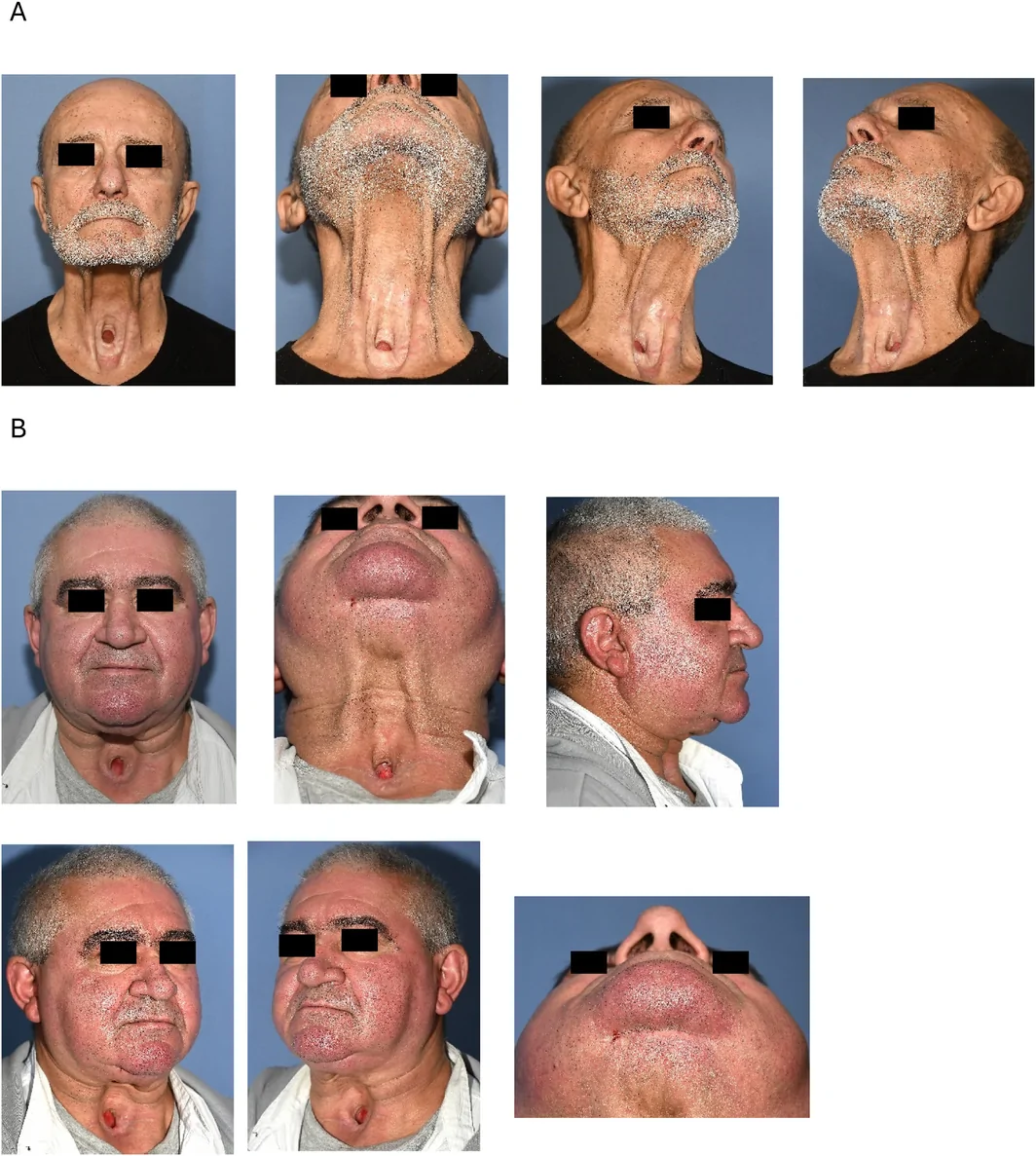
Representative postoperative neck photographs of patients undergoing RESA total laryngectomy. (A) Post‐operative pictures, patient 9, 13 months after RESA. (B) Post‐operative pictures, patient 10, 5 months after RESA. [Color figure can be viewed at wileyonlinelibrary.com]

### 3.3 Survival Outcomes

RESA procedures resulted in an excellent DSS of 100% at a median follow‐up of 15.5 months. Two patients died during follow‐up: Patient 2 died 36 months after RESA partial hypopharyngectomy due to a secondary primary tumor of the floor of the mouth, and Patient 4 died 7 months following RESA from COVID‐19 infection complicated with sepsis. Survival curves are shown in Figure 3A–C.

**FIGURE 3:**
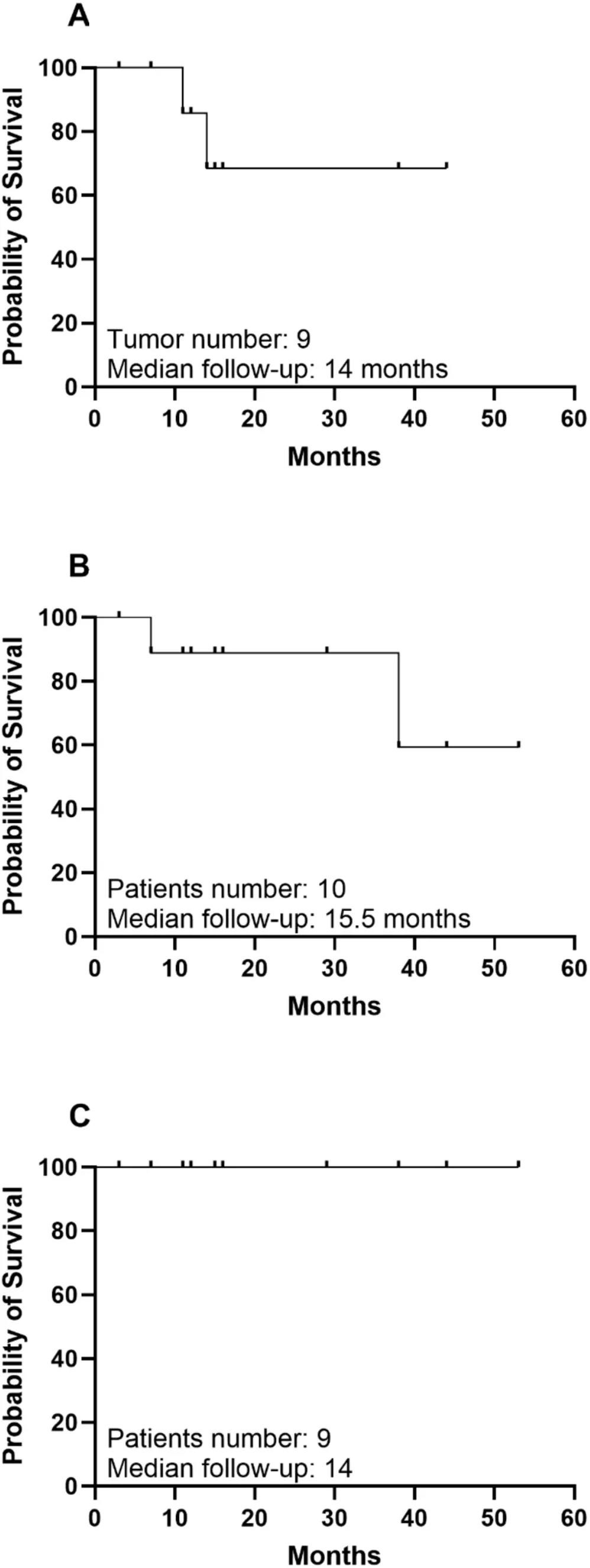
Survival outcomes following RESA: (A) disease‐free survival, (B) overall survival, and (C) disease‐specific survival.

Two patients developed local recurrences: Patient 3 had a recurrence 14 months after RESA, treated with transoral laser microsurgery (TLM), and is currently without evidence of disease; Patient 6 developed a recurrence 11 months after RESA and chose palliative immunochemotherapy at another center, with no further information available.

For patient 3, histopathological findings were favorable, with complete resection of a pT1 tumor and no additional adverse pathological features. However, the tumor was p16‐negative, and the initial presentation was a cT4a tumor occurring in the setting of multiple synchronous primary tumors of the upper aerodigestive tract, including two distinct oral cavity sites and a pulmonary lesion. In this context, a field cancerization phenomenon may account for the subsequent local recurrence despite otherwise favorable histopathological findings.

For patient 6, histopathological analysis confirmed complete tumor resection, with pT4a classification. Despite negative margins, the analysis showed several high‐risk features, including perineural invasion, extensive deep infiltration involving the extrinsic tongue muscles and the arytenoid cartilage, and p16‐negative status. Additionally, reirradiation was not possible, which may have further limited local control.

### 3.4 Functional Outcomes

FOIS scores of patients are presented in Table 4. When comparing preoperative and last available FOIS scores, seven patients (70%) demonstrated improved or stable swallowing outcomes, whereas three patients (30%) showed deterioration. Patient 4 did not recover, as he died due to COVID‐19–related complications 7 months after RESA; Patient 6 developed disease recurrence 11 months after RESA; and Patient 7 developed a second primary pharyngeal tumor 6 months after RESA.

**TABLE 4:** Functional outcomes measured with functional oral intake scale (FOIS).

Patient	Procedure	FOIS score				
		Preop	1 week	1 month	3 months	12 months
Patient 1	Supraglottic partial laryngectomy	4	2	3	3	6
Patient 2	Partial hypopharyngectomy	6	2	3	6	6
Patient 3	Partial base of tongue resection Recurrence 14 months after RESA	5	1	1	3	5
Patient 4	Supraglottic partial laryngectomy	5	1	2	1	Died
Patient 5	Total laryngectomy	1	1	4	1	7
Patient 6	Partial laryngectomy Recurrence 11 months after RESA	4	1	1	2	2
Patient 7	Partial hypopharyngectomy Second primary HNSCC 6 months after RESA	7	1	4	6	1
Patient 8	Total laryngectomy	6	1	5	7	7
Patient 9	Total laryngectomy	4	1	1	7	7
Patient 10	Total laryngectomy	5	1	1	5	F‐U N/A

*Note:* FOIS scores: 1—nothing by mouth, 2—tube dependent with minimal attempts of food or liquid, 3—tube dependent with consistent oral intake of food or liquid, 4—total oral diet of a single consistency, 5—total oral diet with multiple consistencies, but requiring special preparation or compensations, 6—total oral diet with multiple consistencies without special preparation, but with specific food limitations, 7—total oral diet with no restrictions.

Overall, swallowing function was poorest at 1 week postoperatively, followed by a gradual improvement over time (Figure 4). Patients who developed disease recurrence or died exhibited poor outcomes. All total laryngectomy patients recovered swallowing function, with different times of non‐oral feeding (Figure 1). Given the limited sample size, no comparative statistical analysis was performed.

**FIGURE 4:**
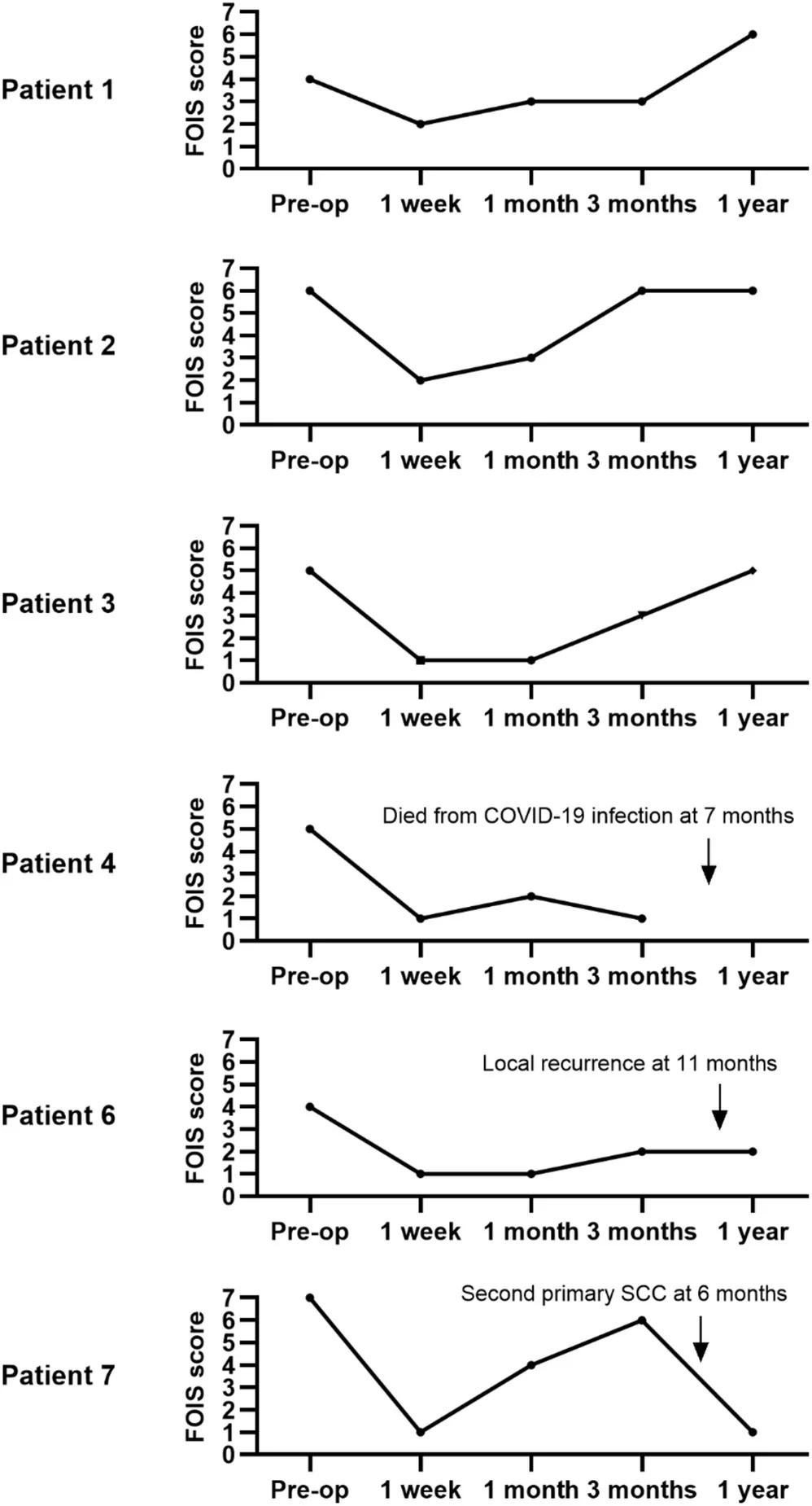
FOIS scores of patients undergoing organ‐preservation RESA.

## 4 Discussion

Robotic‐assisted extended “Sistrunk” approach (RESA) represents a novel minimally invasive surgical paradigm for managing selected head and neck cancers in previously irradiated patients. By providing direct, non‐transoral access to hypopharyngeal and laryngeal structures, RESA enables en bloc resections with favorable margin status, an acceptable complication profile, satisfactory functional recovery, and excellent survival outcomes. Importantly, RESA is not conceived as an extension of transoral robotic surgery (TORS), but rather as an alternative minimally invasive access strategy that broadens the surgical armamentarium for both organ‐preservation procedures and minimally invasive total laryngectomy. In a population frequently requiring multiple interventions for recurrence or second primary tumors, RESA offers a promising salvage option that preserves the principles of minimally invasive surgery while overcoming the intrinsic limitations of transoral exposure.

Limitations in surgical exposure remain a fundamental barrier to transoral approaches, particularly for tumors of the supraglottic larynx and hypopharynx [13, 14, 15]. These constraints are primarily dictated by fixed anatomical relationships—most notably the tongue base, posterior pharyngeal wall, and hyoid bone—which can preclude adequate visualization of deeper laryngeal and hypopharyngeal structures [30, 31]. Prior radiotherapy further exacerbates these challenges through fibrosis and anatomical distortion, often rendering transoral robotic access unfeasible [16]. Although technological advances in transoral platforms may enhance instrumentation, they do not address cases in which exposure itself is fundamentally limited. RESA redefines minimally invasive access by bypassing the transoral anatomical bottleneck through partial central hyoid resection and a median vallecular pharyngotomy, thereby establishing a direct surgical corridor to the laryngeal and hypopharyngeal framework [18, 19].

From a technical standpoint, our findings indicate that RESA is a reliable and reproducible approach, with no required intraoperative conversions to open surgery. Adequate exposure was achieved in all cases, allowing completion of the planned surgical procedures. Negative resection margins (R0) were obtained in all nine oncologic resections, reflecting the ability of the RESA approach to provide adequate exposure in carefully selected patients. These oncologic results compare favorably with published data on salvage TORS, in which a meta‐analysis reported a pooled positive margin rate of 19.4% [24].

Salvage surgery in previously irradiated fields is technically challenging because of tissue fibrosis, altered anatomy, and impaired wound healing, and is associated with increased rates of perioperative morbidity and postoperative complications [32]. Although postoperative complications were not uncommon in this pretreated population, they were consistent with the expected morbidity of salvage surgery in previously irradiated fields. The absence of complications exceeding Clavien‐Dindo stage III underscores the safety profile of the RESA technique and supports its further development (Table 3).

Adequate vascular control is essential during robotic surgery, and the ligation of the external carotid artery has been recommended for TORS [33]. However, this strategy requires a separate open cervical access, which may compromise the minimally invasive concept of RESA. During the procedure, critical vascular structures, including lingual (identified in the Lesser's triangle via the submental incision) and superior laryngeal arteries, can nevertheless be reliably identified and controlled [19]. A meta‐analysis has shown that prior radiotherapy increases the relative risk of post‐TORS hemorrhage, with a pooled postoperative bleeding rate of 13.66% (*n* = 226) [34].

In the present series, postoperative bleeding from the resection bed occurred during the granulation phase (two times day 12, two times day 18). Three events were classified as intermediate according to the Mayo Classification (Clavien–Dindo IIIb), two of which occurred in the first two RESA patients, suggesting a learning‐curve effect, while one event was minor (Clavien–Dindo II) and did not require surgical intervention. Notably, the early bleeding events occurred in patients undergoing partial laryngectomy or pharyngectomy in whom no pexy between the thyroid cartilage and the base of tongue was performed, leaving a larger open and exposed resection bed. Based on this experience and a need to optimize the anatomy for favorable swallowing rehabilitation, a pexy was subsequently introduced in the fourth and fifth patients to reduce the exposed postresection surface, and no postoperative bleeding was observed thereafter among patients undergoing partial laryngectomy or pharyngectomy. In the sixth patient, pexy was not performed as it was deemed unnecessary based on intraoperative clinical assessment. These findings suggest that patients undergoing salvage surgery with large open resection surfaces may be particularly prone to delayed bleeding, and that targeted reconstruction aiming to reduce this surface may mitigate this risk. Notably, a low threshold for operative revision was intentionally adopted during the early phase of technique development, allowing thorough endoscopic assessment and secure hemostasis in all intermediate cases.

A key step of the RESA is the pharyngotomy, which inherently carries a risk of pharyngo‐cutaneous fistula (PCF). Patients undergoing salvage total laryngectomy are known to have a higher risk and severity of PCF compared with those undergoing primary total laryngectomy [35], with reported incidence rates of 43% following primary pharyngeal closure [36]. In organ‐preservation RESA procedures, the pharyngotomy is limited in size and performed in a region with minimal salivary stasis. In this study, no PCF occurred following organ‐preservation surgery. In contrast, the more extensive pharyngeal wall disruption associated with RESA‐assisted total laryngectomy was accompanied by pharyngeal dehiscence in three of four patients. Two cases resolved with conservative management (Clavien–Dindo II), likely facilitated by preservation of the strap musculature, which enabled effective coverage of the primarily closed neopharynx. One patient, initially discharged without signs of dehiscence and permitted oral intake, subsequently developed a delayed dehiscence associated with a cervical abscess requiring endoscopic drainage (Clavien–Dindo IIIb). Notably, no clinically manifest PCF was observed in this patient, nor in the entire series.

Although the number of included patients and the duration of follow‐up were limited, RESA demonstrated encouraging early survival outcomes, with no procedure‐related deaths and a disease‐specific survival (DSS) of 100% following RESA resections; however, these results must be interpreted with caution, given the small sample size, relatively short follow‐up, and strong selection bias. In a retrospective series of 30 salvage TORS procedures, the estimated 2‐year DSS was 93.3% [37]. In the present study, overall survival (OS) was 80% at a median follow‐up of 15.5 months, and disease‐free survival (DFS) was 80% at a median follow‐up of 14 months. By comparison, a meta‐analysis of salvage TORS reported pooled 2‐year OS and DFS rates of 73.8% and 56.1%, respectively [24].

Functional outcomes following RESA were good, with 70% of patients demonstrating preserved or improved swallowing function over time, with swallowing function typically poorest in the early postoperative period and gradually improving thereafter. In a previous study from our group, swallowing function improved in 50% of patients following RESA [19], supporting the consistency of these findings. Comparison with published salvage TORS series remains challenging due to substantial heterogeneity in patient populations, treatment indications, and timing and methods of functional outcome assessment [24, 38, 39].

Case selection for RESA follows a sequential decision pathway. All patients are first evaluated for transoral surgery (TLM/TORS) during diagnostic endoscopy, and RESA is offered when adequate transoral exposure cannot be achieved. Tumor selection requires a disease amenable to either organ‐preserving partial resection (cT1–T3 supraglottic, hypopharyngeal, or tongue base tumors) or total laryngectomy. Contraindications mirror those for standard partial laryngectomy and include tumors invading through the cartilage skeleton when considering total laryngectomy. Patient‐specific factors include fitness for temporary tracheostomy and structured swallowing rehabilitation.

In the presented cohort, transoral exposure failure resulted from radiation‐induced fibrosis and trismus, restricted cervical extension, or unfavorable tongue base anatomy—consistent with established predictors of difficult transoral access. These patients require surgical salvage, yet they are frequently excluded from minimally invasive approaches due to access constraints.

Although evidence regarding the RESA technique is still emerging, and this case series includes a limited number of patients with short follow‐up, the present findings, together with existing evidence, support the further development and careful clinical application of RESA in selected patients at high‐volume centers with substantial TORS experience. Given its minimally invasive access and promising functional and oncologic outcomes, RESA represents a valuable option for salvage and complex head and neck surgery.

## Funding

Schweizerische Nationalfonds (SNF): Sinergia CRSII5_213534 10/23.

## Ethics Statement

The prospective registry was approved by a local bioethics commission (commission cantonale d'éthique de la recherche sur l'être humain [CER‐VD]) no. CHUV_2020_019_RM.

## Consent

All patients signed informed consent for the collection, processing, and publication of their clinical data based on the prospective registry.

## Conflicts of Interest

The authors declare no conflicts of interest.

## Supporting information


**File S1:** Video description.
**Figure S1:** Docking of the da Vinci Xi System through the RESA corridor.
**Figure S2:** Exposure of the right superior cornu of the thyroid.
**Figure S3:** Posterior incision behind the arytenoids in the postcricoid region.
**Figure S4:** Complete mobilization and removal of the larynx.

**Video S1:** RESA Total Laryngectomy.

## Data Availability

The data that support the findings of this study are available from the corresponding author upon reasonable request.
