# Supplementary material for: Robotic‐Assisted Extended Sistrunk Approach (RESA) for Salvage Surgery After Head and Neck Irradiation
Source: Head Neck. 2026 Jun 5;48(11):3180–92. doi: 10.1002/hed.70343 (PMC13653313; doi:10.1002/hed.70343)
Supplement: Supplementary file 1 [file HED-48-3180-s001.docx]

The video presents the robotic phase of RESA total laryngectomy. Following submental incision, creation of a subplatysmal working space, resection of the entire hyoid bone, and vallecular pharyngotomy, the da Vinci Xi robotic system is docked through the RESA corridor (Supplementary Figure 1). The procedure begins in the vallecular region with exposure of the anterior border of the thyroid cartilage and bilateral superior cornua (Supplementary Figure 2). A posterior incision is then made behind the arytenoids through the post-cricoid region (Supplementary Figure 3) – the tumor is intralaryngeal, and both piriform sinuses are preserved after being released. Circumferential mobilization of the larynx is subsequently performed, including the thyroid and cricoid cartilages. The right superior cornu of the thyroid cartilage is divided using conventional surgical scissors, allowing further mobilization of the thyroid cartilage. The operative plane then connects inferiorly with the tracheal plane on the right and medially. Subsequently, the left superior cornu – the remaining point of laryngeal attachment – is dissected and divided using scissors. Complete mobilization and removal of the larynx are achieved (Supplementary Figure 4).

Supplementary Figure 1. Docking of the da Vinci Xi System through the RESA corridor.

Supplementary Figure 2. Exposure of the right superior cornu of the thyroid.

Supplementary Figure 3. Posterior incision behind the arytenoids in the postcricoid region.

Supplementary Figure 4. Complete mobilization and removal of the larynx.
